# COVID-19: A Rural US Emergency Department Perspective

**DOI:** 10.1017/S1049023X20001417

**Published:** 2020-11-04

**Authors:** Angelika Underwood

**Affiliations:** Emergency Room, Bassett Healthcare, Cooperstown, New York USA

**Keywords:** COVID-19, COVID-19 testing, emergency department, emergency room, PPE, COVID-19, coronavirus disease 2019, ED, emergency department, EMS, Emergency Medical Services, PPE, personal protective equipment

## Abstract

Several aspects led to the poor control of the coronavirus disease 2019 (COVID-19) outbreak in the US from a rural emergency department (ED) perspective. These include US residents’ attitude towards political involvement in health and civil rights; lack of enough testing kits and rapid test results, or not available at all; and personal protective equipment (PPE) shortages. These obstacles related to medical supplies and resources, and lack of coordinated approach to the pandemic in the US, are important information for retrospective disaster research to understand study limitations, extrapolate accurate and valid data, and for other countries to understand how and why the US had higher numbers of COVID-19 cases and deaths compared to other countries.

Coronavirus disease 2019 (COVID-19) spread through the United States (US) and closed down most states in mid-March 2020. As of the time of writing this Editorial, the US has had 6.8 million test-positive cases of COVID-19, with approximately 200,000 deaths, placing the US highest on the list of COVID-19 cases and deaths.^1^ This is a discussion of the spread of COVID-19 in the US from the perspective of a rural emergency department (ED) provider.

There are several factors leading to poor US control of COVID-19. US resident attitudes toward political involvement in health and civil rights are the main reason for many problems with control of the pandemic in the US.

Also, testing within the US has not been made universally available. Initially when the outbreak hit, there were not enough tests for organized population testing. People were told to self-isolate for 14 days at home if they experienced symptoms, unless severely ill, at which point they were instructed to present to an ED. If patients presented to out-patient clinics or convenient health care sites, they initially had an influenza test and respiratory antibody laboratory panel with the theory that if a patient was positive for anything on the respiratory panel or for influenza, they probably didn’t have COVID-19, based on early studies indicating the rate of respiratory co-infection was very low.^2-4^ Approximately two to three weeks after the initial outbreak, testing media was running low for the viral respiratory panel and that was discontinued with symptomatic patients then only tested for influenza. If influenza negative and a patient was symptomatic, they would be sent home with presumed COVID-19 with instructions to self-isolate for 14 days unless symptoms of respiratory distress worsened. Only the very ill ED patients could be tested for COVID-19, and no testing was available in the clinic or convenient care settings. The local Departments of Health had to be called for all COVID-19 suspected patients to approve of the COVID-19 testing. This health department approval was based upon whether a person traveled to a high-risk pandemic outbreak area overseas, such as China. This type of testing restriction resulted in delay for COVID-19 screening, even after wide-spread US community spread was recognized. This lasted almost two weeks, after which testing became more available with test specimens required to be sent to centralized local state testing sites, with one- to two-day delays in reporting results for symptomatic patients, and five to seven days for asymptomatic patients. This delay in turnaround allowed many people to be in contact with a possible infected COVID-19 patient while waiting for results. In addition, COVID-19 infected persons may have been contagious two days prior to becoming symptomatic.^5^ If a patient was admitted to a hospital, delays in receiving results for COVID-19 testing contributed to excess use of resources, such as personal protective equipment (PPE) and negative pressure rooms when often the patient was negative for the virus.

All patients admitted to the hospital after April 2, 2020 were tested for COVID-19 regardless of whether they had symptoms. The test itself was operator dependent. For example, a nasopharyngeal swab needs to go quite far up in the nasal cavity for correct specimen collection and some personnel obtaining a sample did not go up far enough, or the patient wouldn’t allow them to go far enough, telling them to stop or pulling away. In addition, testing was not possible for patients with nasal deformities such as a deviated septum.

World-wide PPE shortages have also been a problem. To address this in the US, action was taken to reuse and to extend the use of PPE to attempt to supply enough N95 respirators (face masks). The US Centers for Disease Control and Prevention (CDC; Atlanta, Georgia USA) recommendation during this stage of the pandemic was reuse PPE up to five times, or extend use up to eight hours.^6^ Initially, N95s were worn for three days, and then as a last resort, saved in case all N95 masks were depleted. On April 2, 2020, that recommendation was stretched out to reusing the masks for seven days, then sterilizing and wearing again for seven days. After a few months, more and various types of N95 masks became available, and staff were refitted to wear these various N95 disposable masks or full- and half-face mask reusable respirators.

Early in the pandemic, there was a push for early, aggressive endotracheal intubation of sick patients with respiratory symptoms. Patients who normally would be managed with a trial of non-invasive positive pressure ventilation (ie, continuous positive airway pressure [CPAP] or bilevel positive airway pressure [BIPAP]) were not given a non-invasive trial due to concerns of spreading the virus into the room environment. Endotracheal intubation utilizes a closed air circuit and was considered safer for staff. But many of these patients were put at risk as they had prolonged intubation times, often up to one month.

Emergency Medical Services (EMS) were affected by COVID-19 as well as EDs. As of April 3, 2020, many out-of-hospital EMS providers adopted a protocol stating any cardiac arrest would not be taken to an ED and rather pronounced dead on scene, unless return of spontaneous circulation was obtained.

US residents, in general, felt their civil and personal rights were being violated by government and public health providers. No universal law went into effect in “shutting down” the country. All states were allowed to make their own rules regarding mask wearing enforcement, closing businesses, and for social distancing laws. Mandatory mask wearing took time to be enforced. Some local counties adopted social distancing and shutting down businesses, while other counties in the same state were open as normal. When US residents were told to stay home for two weeks or wear masks, some refused with the attitude that no one can make them do anything “violating” their rights. Even though masks are required in business locations, some people still refused to wear them without consequences. Also, some people blamed the presidential election as a reason for all the rules, such as businesses not being able to open normally. As of mid-September 2020, six months after the pandemic hit the US, they are still waiting up to five days for results after patients are admitted to hospitals and many patients are discharged back into the community before COVID-19 results return. While many places have point-of-care tests available for COVID-19, there remains certain areas which still can’t get the test, or have a limited number of test kits available, and can’t get more at this time. In addition, N95 masks supply is unreliable and many are reusing a mask for three days.

There are many things hospitals have done well with quick assembly of outside screening tents, integrating telehealth, and increasing negative pressure airflow room; but overall, there were many obstacles to quickly eliminating COVID-19. Most often, obstacles were related to medical supplies and resources, as well as lack of coordinated approach to the pandemic. Many countries had similar problems with much better success compared to the US, but their cultures are more accepting of shutting down their country to protect the community.

Finally, disaster research is difficult, and many papers use retrospective data, making it difficult to seek out the study limitations and extrapolate accurate and valid data. Hopefully, all can learn from this pandemic to better prepare for the next pandemic.
